# HIV Social-network intervention more effective in older populations in Kenya

**DOI:** 10.1186/s12889-024-20315-0

**Published:** 2024-11-09

**Authors:** Daniel E. Zoughbie, Dillon Huddleston, Kathleen Watson, Eric L. Ding

**Affiliations:** 1 Department of Public Health, New England Complex Systems Institute, 277 Broadway, Cambridge, MA 02139 USA; 2 Social Network Research Group, Microclinic International, 548 Market St. Ste 63776, San Francisco, CA 94104-5401 USA; 3 Institute of International Studies, University of California at Berkeley, 215 Philosophy Hall Berkeley, Berkeley, CA 94720-2308 USA; 4 Stanford University School of Medicine, Stanford, CA 94720-2308 USA

**Keywords:** HIV/AIDS, Kenya, Patient retention, Interaction, Mediation, Intent-to-treat

## Abstract

US President’s Emergency Plan for HIV/AIDS has been credited with saving 25 million lives in sub-Sahara Africa and, as such, constitutes a preeminent US foreign policy achievement of the twenty-first century. However, the implementation of effective HIV/AIDS pharmacological interventions remains a challenge in rural Kenyan communities. Of particular importance are patient retention and care engagement and their interaction with age disparities that are sensitive to different socioeconomic contexts, as well as time-in-treatment. For the first time, we perform an intermediation and triple interaction intent-to-treat secondary analysis on a social network-based randomized controlled trial. We hypothesize that the temporal interactions of critical demographic features with a treatment/control indicator variable may significantly explain patient retention and that these results are intermediated by social network phenomena. We find that not only does extended time-in-treatment significantly improve primary outcomes, but the threefold interaction along with age and treatment itself is sufficiently flexible to fit the data remarkably well without unnecessary elaboration, an effect that is mediated via internalized stigma. This strongly suggests that patient retention varies by age group. Rather than deploying one-size-fits-all solutions, foreign and public policymakers should invest in research that considers how interventions might be optimized for different ages.

**Trial registration **Clinical Trial Number. NCT02474992 (note: the main trial report was published here 10.1371/journal.pone.0255945.) Date of submission: June 6, 2015.

## Introduction

Despite recent advances in HIV/AIDS research, the implementation of effective disease management strategies remains a topic of continued interest [8], especially in less commonly studied communities. A key challenge involves keeping patients engaged in lifesaving care, which not only enhances their chances of survival but also decreases the likelihood of transmission and ART resistance [22, 29, 37, 38].

Kenya’s National AIDS Strategic Plan lists one of its strategic actions as engagement, i.e., reducing interruptions in care and death and increasing ART initiation (National HIV Strategic Plan, 2021–2026). Due in large part to international aid programs dubbed the "Lazarus Effect," care engagement efforts have made significant progress over the years [5–7, 53], notably, the PEPFAR program is estimated to have saved 25 million lives [23]. Yet formidable implementation science challenges remain [18, 24, 51].

Hallmark features of HIV/AIDS are age disparities that are sensitive to different socioeconomic contexts [10]. For example, in the United States, new HIV infections occur more among men (83%), men who have sex with men (MSM) (68%), and between the ages of 13–34 (57%) (HIV Surveillance Report, 2020). By contrast, MSM accounts for only 15.2% of total new HIV infections in Kenya [39], disproportionate new infections occur in men aged 20–29 and women aged 15–24 [46]. Evidence from Ethiopia suggests that adolescent women bear the greatest infection burden [25].

Age is not only known to interact regarding HIV/AIDS transmission dynamics [26] but also to affect disclosure, social support, and care engagement, both globally and in Sub-Saharan Africa [4, 10, 12, 17, 19, 30, 31, 33, 36, 42, 43, 46, 49, 50, 56–58]. Time is also an important variable, with untreated AIDS, patients generally will not survive beyond three years [55]. The Joint United Nations Programme on HIV/AIDS country statistics show just how important time, age, and gender are to understanding the HIV epidemic [45].

In Kenya’s Homa Bay County, HIV rates are 4.5 times the national average, with age and gender as significant risk factors [1, 20, 40]. Here, poverty rates are estimated at 77.9% compared to 55.2% in Kenya more generally [41]. Mfangano Island, situated within Homa Bay County and home to approximately 20,000 people, has one of the highest combined prevalences of HIV and poverty in the world. Eighty-seven percent of the population engages in "hand-to-mouth" subsistence farming [9].

We conducted an intermediation and interaction analysis for the first time on a Microclinic ("Kanyakla" or "team" in Dhuluo) Social Network-based randomized controlled trial on Mfangano Island [28]. As recommended by the US Food and Drug Administration, we hypothesized that the temporal interactions of critical demographic features with a treatment/control indicator variable may significantly explain longitudinal outcomes (remaining in treatment or not). We further hypothesized that these results were intermediated by social network phenomena.

## Methods

### Study population

The research was carried out on the islands of Mfangano, Takawiri, Ringiti, and Remba, located in Homa Bay County, Kenya, where the HIV prevalence among adults aged 15 to 49 is estimated to be 21%. Eligibility for participation was extended to adult patients aged 18 or older at any of the nine rural, government-operated health centers within the study’s designated area, provided they had missed a clinic visit by more than three days during the enrollment period. This enrollment period spanned from August 2015 to February 2016, and the collection of data continued until February 2018. Criteria for exclusion from the study included prior participation in a microclinic pilot study at any of these clinics, residence outside the designated study area, or intentions to relocate from the study area within the subsequent six months. The study received approval from both the Ethical Review Committee of the Kenya Medical Research Institute and the Human Research Protection Program of the University of California, San Francisco. The protocol for this study was officially registered on ClinicalTrials.gov under the identifier NCT02474992, and written informed consent was secured from all participants before their enrollment. The trial analysis was previously published, showing that the intervention was effective in improving the secondary but not primary endpoint. The CONSORT diagram and accompanying checklist are published as follows: [28].

### Microclinic social network

The intervention utilizing microclinics involved creating small groups comprising 5 to 10 individuals, including close family, friends, and other members of the participant’s social support network, regardless of their HIV status. These microclinic groups did not dispense antiretroviral therapy but rather augmented clinic-based care with community-based support for individuals living with HIV. In the local Dholuo language, these groups were referred to as “kanyaklas,” translating to “team” or “together.” At the initiation of these groups, each member received individual HIV counseling and testing. Subsequently, a Community Health Worker (CHW) was appointed as a facilitator for each group, and they led eight bi-weekly sessions held at locations chosen by the group. The curriculum of the microclinic covered eight key topics: 1) program overview and confidentiality within the group, 2) local epidemiology and prevention of HIV, 3) fundamentals of HIV treatment, 4) group support for adherence to HIV medication and engagement in healthcare, 5) local beliefs surrounding HIV, herbal treatments, and nutrition, 6) group support in addressing HIV-related stigma, 7) collective disclosure of HIV status within the group, and 8) a debriefing session focusing on group disclosure and ongoing group support. Results presented here are also consistent with observational and randomized trials showing improvements in other chronic disease management programs that utilized the Microclinic social network model in the following different geographical and socio-economic contexts: Jordan, Lebanon, Palestine, and Kentucky [13–15, 44, 48, 59, 60].

### Mediators

Earnshaw stigma scale: We evaluated mean scores on the HIV-associated stigma scale developed by Earnshaw et. al. [16] to assess overall stigma, as well as internalized, anticipated, and enacted stigma at baseline and 12 months. Specifically, differences between means of the following variables on the Ernshaw stigma scale from baseline to the end of the study were considered as mediators: Internalized, anticipated, enacted, and overall stigma; emotional, material, clinic visit, and adherence support; and the number of (close) support network members whom the participant knows or who knows the participant’s HIV status.

### Outcome

Patient outcomes were classified as either continuously in care, 90-day disengagements from care, death, and transfer to a facility outside the region. Pre-specified primary outcomes were disengagement from care and ‘time in care’. Our primary disengagement outcome was defined as the time to the first instance of a 90-day absence from any discernable clinical care during 12 months of follow-up. Time in care was defined as the proportion of follow-up time spent adhering to clinic visit schedules over 12 months of follow-up [27].

We calculated gaps in care by determining the number of days from a missed clinic visit until return to any clinic within Homa Bay County. Participants were censored on the date of death or transfer to a facility outside Homa Bay County. Thus, 90-day disengagement indicates missing an appointment by at least 90 days and not known to have first transferred to another facility or died. Time in care is the proportion of follow up time that a participant adhered to clinic appointments and was calculated as [(total follow up time)–(sum of gaps in care)]/(total follow up time).

### Statistical analysis

Using intention-to-treat analysis, we compared the time series of patient outcomes between study arms using multivariate linear models over the entire study period for each outcome accounting for interactions between 1) age and treatment effect and 2) gender and treatment effect.

The intent-to-treat analysis considers the following (null) multivariate regression models:

$${y}_{ij}={\beta }_{0}+{\beta }_{1}{t}_{ij}+{\beta }_{2}{\delta }_{i}+{\beta }_{3}{t}_{ij}{\delta }_{i}+{\beta }_{4}{a}_{i}+{\beta }_{5}{t}_{ij}{a}_{i}+{\beta }_{6}{\delta }_{i}{a}_{i}+\gamma {\prime}{X}_{i}+{\varepsilon }_{ij}$$

$${y}_{ij}={\beta }_{0}+{\beta }_{1}{t}_{ij}+{\beta }_{2}{\delta }_{i}+{\beta }_{3}{t}_{ij}{\delta }_{i}+{\beta }_{4}{g}_{i}+{\beta }_{5}{t}_{ij}{g}_{i}+{\beta }_{6}{\delta }_{i}{g}_{i}+\gamma {\prime}{X}_{i}+{\varepsilon }_{ij}$$

**Fig. 1:**
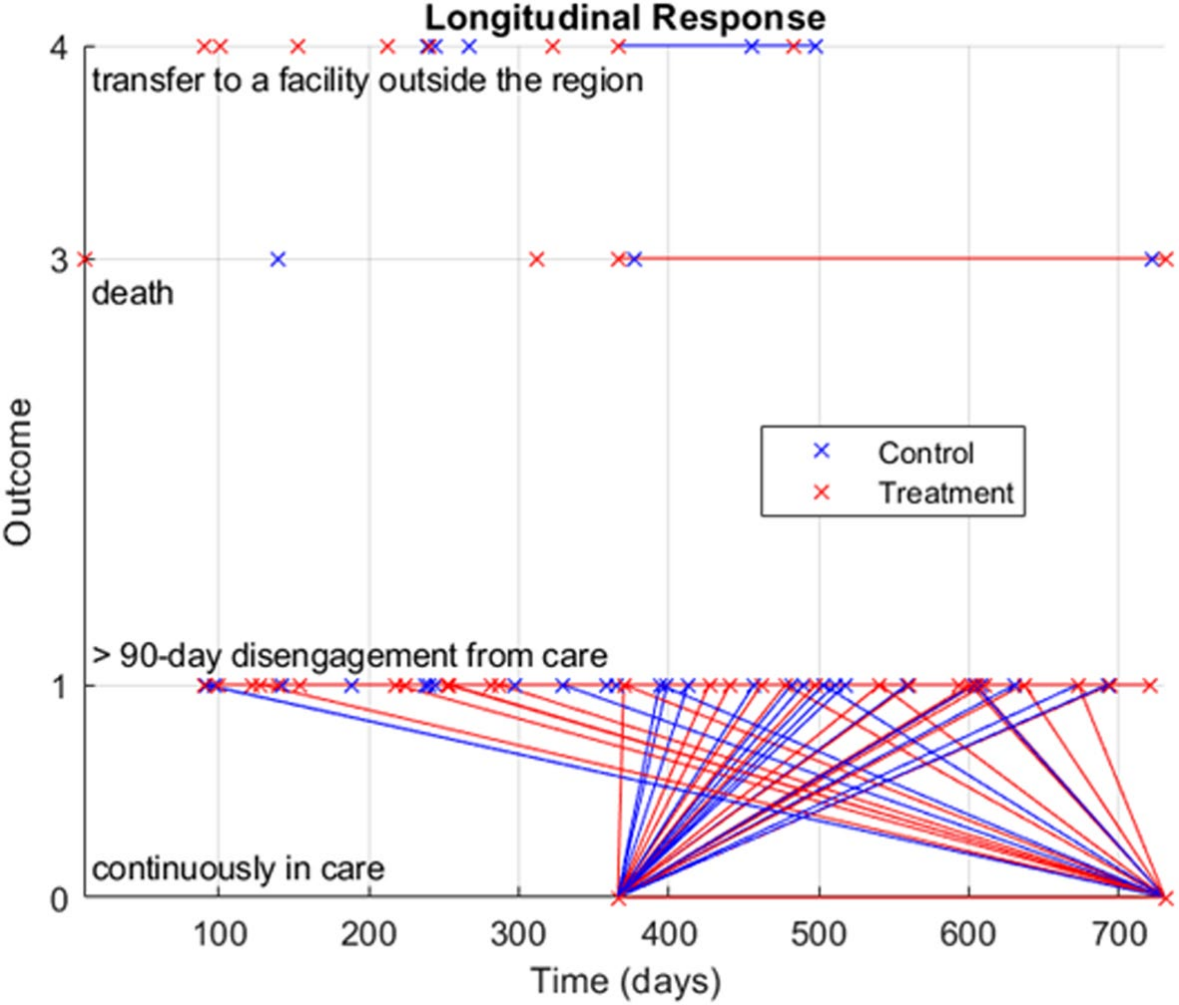
Longitudinal outcomes by treatment status

Here, ${y}_{ij}\in \{\text{0,1},\text{3,4}\}$ denotes the outcomes (respectively continuously in care, 90-day disengagements from care, death, and transfer to a facility outside the region) of participant *i* at the time ${t}_{ij}$ (there being three measurements per participant, generally taken at differing times, $j\in \{\text{1,2},3\}$), plotted in Fig. 1; ${\delta }_{i}\in \{\text{0,1}\}$ denotes control (0) or treatment (1) group; ${X}_{i}$ is a vector of controls (years since HIV diagnosis and first clinic enrolment and indicators for microclinic program participation, antiretroviral therapy status, and HIV status disclosure outside the clinic); ${a}_{i}\in \{\text{18,25,50}\}$ indicates age group (respectively, 18–24, 25–49, and 50 years or older); ${g}_{i}\in \{\text{0,1}\}$ denotes female (0) or male (1); and ${\varepsilon }_{ij}$ is multivariate normal with mean zero. An interaction of the basic treatment effect, ${t}_{ij}{\delta }_{i}$, with age group and gender are each hypothesized via the following nested specifications of the null model:

Age group by treatment by time in treatment triple interaction:

$${y}_{ij}={\beta }_{0}+{\beta }_{1}{t}_{ij}+{\beta }_{2}{\delta }_{i}+{\beta }_{3}{t}_{ij}{\delta }_{i}+{\beta }_{4}{a}_{i}+{\beta }_{5}{t}_{ij}{a}_{i}+{\beta }_{6}{\delta }_{i}{a}_{i}+{\beta }_{7}{t}_{ij}{\delta }_{i}{a}_{i}+\gamma {\prime}{X}_{i}+{\varepsilon }_{ij}$$

Gender by treatment by time in treatment interaction:

$${y}_{ij}={\beta }_{0}+{\beta }_{1}{t}_{ij}+{\beta }_{2}{\delta }_{i}+{\beta }_{3}{t}_{ij}{\delta }_{i}+{\beta }_{4}{g}_{i}+{\beta }_{5}{t}_{ij}{g}_{i}+{\beta }_{6}{\delta }_{i}{g}_{i}+{\beta }_{7}{t}_{ij}{\delta }_{i}{g}_{i}+\gamma {\prime}{X}_{i}+{\varepsilon }_{ij}$$

## Results

In the intervention group, 72% of the participants, amounting to 111 individuals, took part in a microclinic group, with participation rates being 70% for men and 73% for women. Additionally, 3% of participants in the control group, specifically four individuals, were invited to join the microclinics of other participants, resulting in their participation in a microclinic group as well. All data analyses were performed following an intention-to-treat approach. Overall, 50 microclinic groups were established, encompassing a total of 485 participants, with each group varying in size from 5 to 10 members.

### Interaction analysis

The analysis revealed that for age group, the interaction with treatment was significant, with a *p*-value < 0.0001. Indeed, the original insignificance of the estimates for ${\beta }_{2}$ through ${\beta }_{6}$ are seen to be replaced by significance (to more than five decimal places) along with that for ${\beta }_{7}$; and a likelihood ratio test rejects the null in favor of this alternative model at the 5% level (and indeed, to more than four decimal places). This indicates that the effect of (time-in-) treatment interacts strongly with the age group, to the extent that without such interaction, no significance is observed beyond a time trend; whereas once the interaction is separately specified, all other terms are highly significant. Specifically, the triple-interaction of age group with (time-in-) treatment (captured by ${\beta }_{7}$) is seen to be small but negative; associated with one day of treatment and a transition from the youngest to the intermediate or the latter to the eldest age group is a -6.32E-5 change in outcome; i.e., reengagement in care.

Conversely, for gender, the model showed an opposite trend; the gender interaction with treatment was not significant, and the likelihood ratio test did not support the alternative gender model over the null, indicating that gender did not have a significant interaction effect with the treatment. This includes a non-significant estimate of the triple interaction with (time-in-)treatment and failure of a likelihood ratio test to reject the null in favor of the alternative gender model.

Table 1 tabulates trial participants’ baseline characteristics.

**Table 1:**
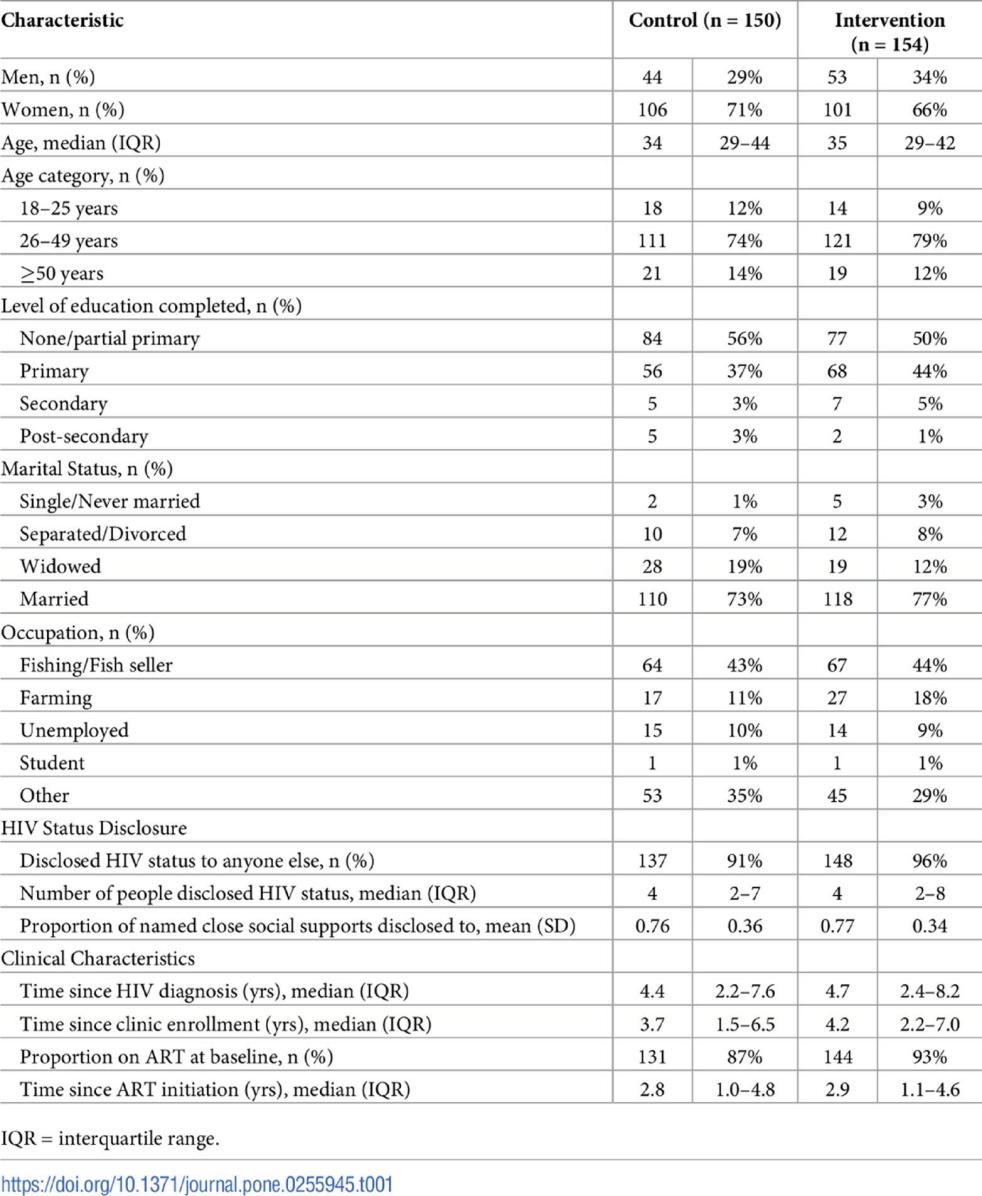
Baseline characteristics of enrolled participants

Table 2 tabulates the coefficient estimates (significance at the 5% level being indicated by italicization), along with likelihood ratio (LR) test *p*-values for the two alternative models vs. the null.

**Table 2:** Null vs. triply interacted model coefficient estimates

Model	$\beta_0$	$\beta_1$	$\beta_2$	$\beta_3$	$\beta_4$	$\beta_5$	$\beta_6$	$\beta_7$(interaction)	LR *p*
Age Group (Null)	*1.25*	*-0.0020*	-0.093	-0.0051	2.86E-5	7.39E-6	0.0023	-	-
Age Group	*1.93*	*-0.0032*	*-0.96*	*-0.034*	*0.0016*	*5.90E-5*	*0.037*	*-6.32E-5*	*2.04E-5*
Gender (Null)	*1.17*	*-0.0019*	-0.074	-0.077	6.54E-5	*1.43E-4*	0.050	-	-
Gender	*1.18*	*-0.0019*	-0.096	-0.11	1.07E-4	2.00E-4	0.098	-9.60E-5	0.59

Except as tabulated below, those for the controls, $\gamma$, were nonsignificant for all models (Table 3).

**Table 3:** Null vs. triply interacted secondary outcome coefficient estimates

Model	Time since clinic enrollment	Clinic ART status	Patient ART status
Age (Null)	*-0.014*	*0.16*	*-0.12*
Age	*-0.015*	*0.16*	*-0.12*
Gender (Null)	*-0.012*	*0.15*	*-0.12*
Gender	*-0.012*	*0.15*	*-0.11*

As such, the null model is rejected in favor of the age- rather than gender-based alternative, and higher-order interaction between these crucial demographic variables need not be considered. Of course, the crucial difference between the null model and this alternative within which it nests is the additional, treatment-dependent addend, ${\beta }_{7}{t}_{ij}{\delta }_{i}{a}_{i}$, which captures the extent to which treatment outcomes are explained by twofold interactions between age and time. The likelihood ratio test for this model specification hypothesis, which yields a *p*-value of approximately zero, indicates significant evidence for the rejection of the null model in favor of the alternative, i.e., that *treatment outcomes are significantly explained by twofold interactions between age and time*. In other words, treatment status significantly explains differential participant outcomes when interacting with age and time.

**Fig. 2:**
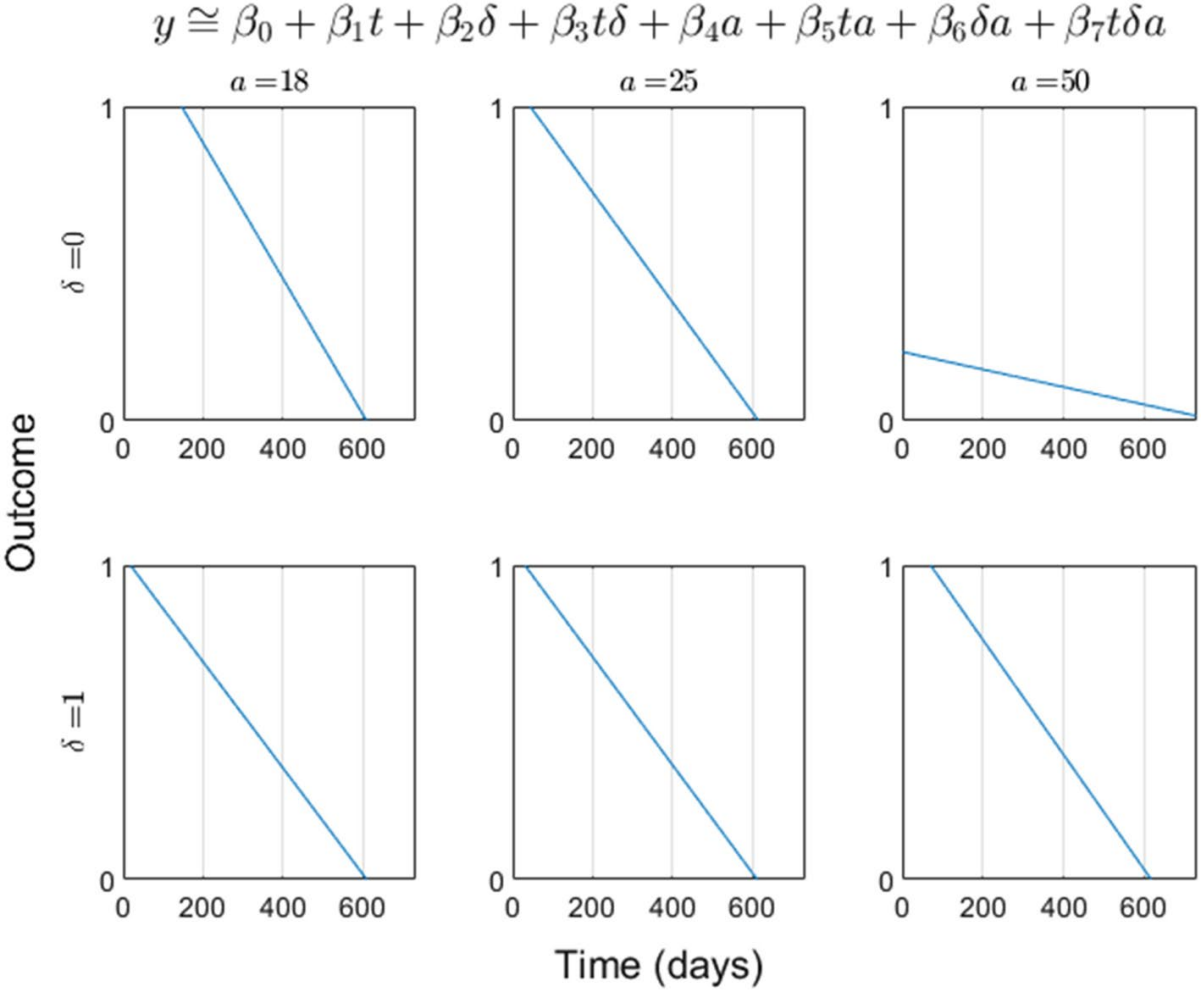
Longitudinal regression fit by treatment status and age group

Concerning treatment efficacy by age group, this is not immediately clear from the tabulated regression coefficients; however, plotting the model predictions over time for unique age groups and treatment statuses clarifies matters: Interpreting the time-in-treatment effect upon outcome via the (approximate, though clearly nearly linear due to differences in coefficient estimate orders of magnitude) slope, slight differences between the top and bottom row (respectively corresponding to control and treatment) are apparent in the first two columns, corresponding to age groups [18,25) and [25,49); however, the improvement appears to be an order of magnitude greater in the third column which corresponds to the over-50 age group. As such, it is indeed the case that *time-in-treatment markedly improves treatment outcomes for the over-50 age group*. In fact, it appears that for the [18,25) ([25,49)) age group, the time-in-treatment effect actually (slightly) *worsens* outcomes under treatment vs. control, a surprising finding with clinical implications that also merits further exploration (Fig. 2).

It should be noted that entries with outcomes three or four were dropped since right-censoring them [a la Hickey et al. [28]] yields only isolated points on which longitudinal analysis cannot be performed. This assumes [again a la Hickey et al. [28]] that baseline outcomes all zero are not imposed; in case they are, longitudinal analysis or other regression on outcomes are precluded. Additionally, conceptually, doing so is contrary to the notion of predictors determining outcomes since, at baseline, the experiment is designed only to intake one outcome (zero).

The significant control estimates are remarkably consistent across all models, both null vs. alternative and gender vs. age group. This is consistent with the result of Hickey et al. [28], which found significant impacts on the *secondary* rather than the primary outcomes (without age or gender effect modifications as in the present study). Likewise, an analogous mediator analysis for the alternative gender model finds significant mediation by the same secondary outcome (the difference between baseline and study end average internalized stigma on Earnshaw stigma scale) of the three-fold (time-to-)treatment gender interaction impact on the primary outcome, insignificant such as the latter is.

### Mediation analysis

**Fig. 3:**
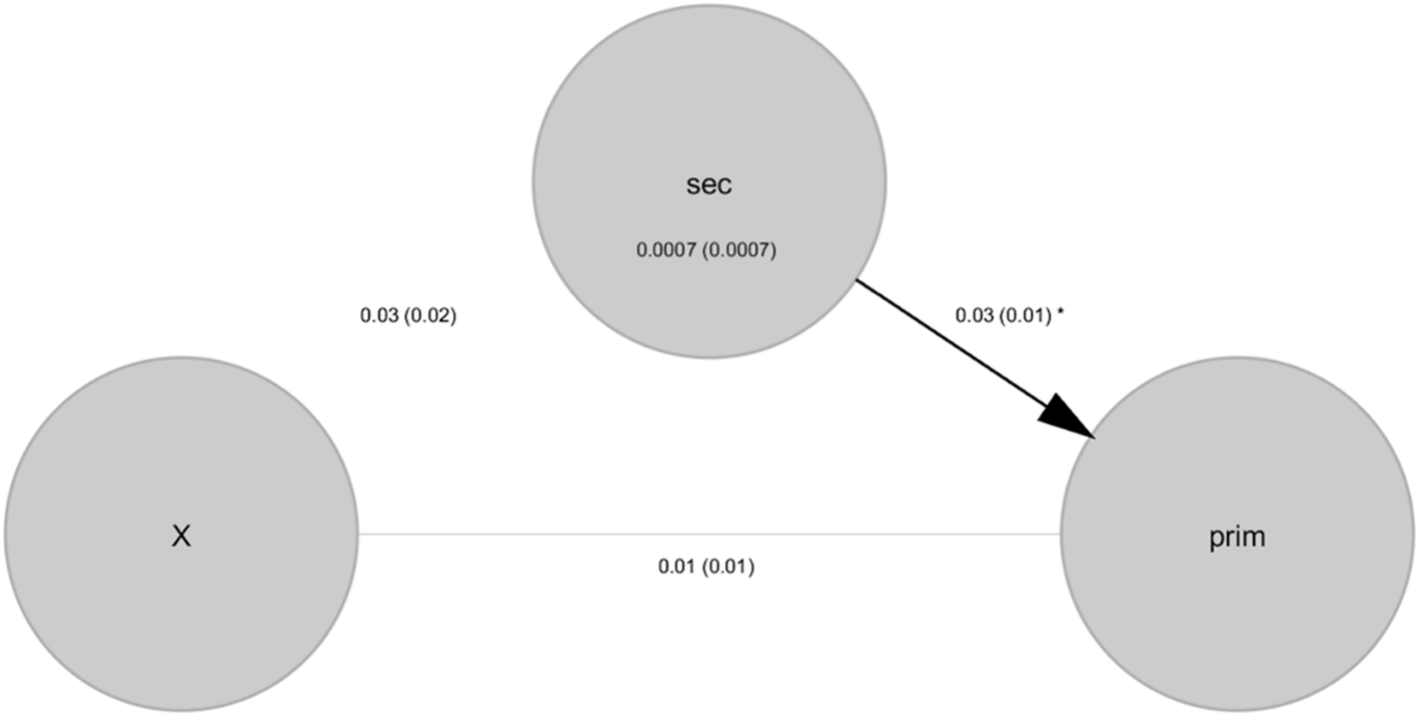
Mediator analysis graphical model: The treatment-dependent, twofold interaction between age and time (‘X,’ denoting ${t}_{ij}{\delta }_{i}{a}_{i}$) predicts the primary outcome (‘prim,’ denoting ${y}_{ij}$) via the corresponding alternative model; which is significantly mediated by the secondary outcome (‘sec,’ denoting the difference between baseline and study end average internalized stigma on Earnshaw stigma scale)

**Fig. 4:**
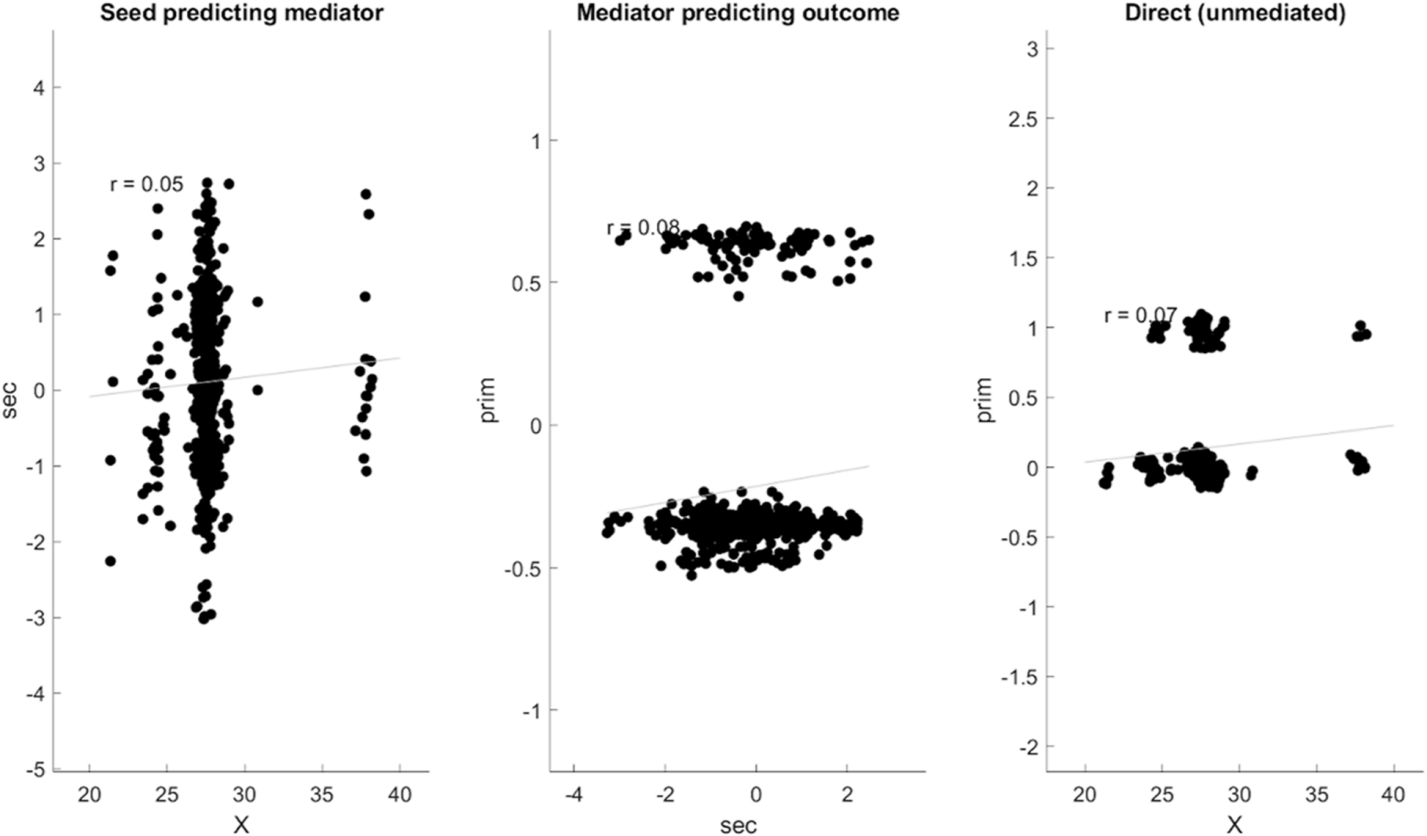
Mediator analysis regression scatter plots

Furthermore, a mediation analysis using the Matlab toolbox of Wager et al. [54] found that this effect is significantly mediated (*p* = 0.026) by the *difference between mean internalized stigma on the Ernshaw stigma scale from baseline to the end of the study*. [Also considered and rejected as mediators were similar differences in anticipated, enacted, and overall stigma; emotional, material, clinic visit, and adherence support; and the number of (close) support network members whom the participant knows or who knows the participant’s HIV status.] That is, the treatment-dependent, twofold interaction between age and time (‘X,’ denoting ${t}_{ij}{\delta }_{i}{a}_{i}$) predicts the primary outcome (‘prim,’ denoting ${y}_{ij}$) via the corresponding alternative model; which is significantly mediated by the secondary outcome (‘sec,’ denoting the difference between baseline and study end average internalized stigma on Earnshaw stigma scale). Plotted in (Figs. 3 and 4) are a causal diagram and prediction plots summarizing these results:

Table 4 tabulates additional results of the (single-level model) mediation analysis.

**Table 4:** Mediator analysis coefficient estimates

	X → sec	sec → prim	Unmediated X → prim	X → prim	Mediated X → prim by sec
Coeff	0.03	*0.03*	0.01	0.01	0.00
STE	0.02	*0.01*	0.01	0.01	0.00
t (~N)	1.22	*2.23*	1.86	1.95	1.00
Z	1.22	*2.22*	1.85	1.95	1.00
p	0.22	*0.03*	0.06	0.05	0.32

## Discussion

Overall, the complementary nature of the present results and those of Hickey et. al. [28] indicate that the underlying treatment program was successful in secondary endpoint improvements among the general study population; but was successful only among the > 50 year-old population for the primary endpoint. Additionally, we link the two analyses via mediation of the primary endpoint by the secondary. In particular, not only does the age (null) model indicate that extended time-in-treatment significantly improves primary outcomes, but the threefold interaction along with age and treatment itself is sufficiently flexible to fit the data remarkably well without unnecessary elaboration: This term alone is highly significant, along with all lower-order ones which are not in the null model. Though this does not immediately prescribe a unique policy response, it does strongly suggest that the longitudinal treatment outcomes vary by age group. This finding is therefore highly important for clinicians looking to optimize and personalize the provision of care among populations of different ages.

Our study suggests that not only does treatment status significantly explain differential participant outcomes when interacting with age and time. The mechanism by which this occurs is significantly (and exclusively among the considered mediators) mediated via internalized stigma, which is targeted for reduction by the Microclinic social network intervention, a primary design feature of the latter. Social stigma is by definition a social network phenomenon whereby individuals with HIV/AIDS are made to feel socially ashamed amongst their peers and family. Utilizing social networks to destigmatize HIV/AIDS, to help affected individuals feel as if they are not alone, reduces social anxiety, opening the pathway to care engagement and retention.

In particular, since disclosure and stigma reduction have been proposed as factors associated with higher care retention [3, 32], this is a remarkable finding, suggesting that further studies should investigate generalizable causal mechanisms through which social network effects protect against disengagement. Because the study was randomized, we believe the emotional (psychosocial) mediation effects of the Microclinic Social Network Model are unlikely to be confounding or reverse causation (homophily) and should be considered carefully alongside other causal evidence.

These results are also consistent with a prior quasi-experimental Microclinic social network study, which was launched on Mfangano Island using a more intensive intervention program and showed greater improvements in care engagement [27]. Results presented here are also consistent with observational and randomized trials showing improvements in other chronic disease management programs that utilized the Microclinic social network model in the following different geographical and socio-economic contexts: Jordan, Lebanon, Palestine, and Kentucky [13, 15, 44, 48, 59, 60].

This study has several important limitations. While the primary and secondary endpoints were defined a priori, the time-age-treatment interaction analysis was not included in the original protocol and is, therefore, secondary to the main trial analysis [28]. Nevertheless, we believe it has high credibility.

The hypothesis of age and gender as variables whose three-fold interactions with time-in-treatment and treatment itself add significant explanatory power for corresponding longitudinal outcomes are eminently justifiable via multiplestrands of literature. Cochrane and the National Institutes of Health encourage consideration of specific demographic populations in analysis [35]. In the absence of standardized factors for such (interaction) purposes with RCT data, Christensen, Bours, and Nielsen [11] emphasize that the U.S. Food and Drug Administration (FDA) mandates analysis via both sex [21] and age, as well as race [52]. In particular, these guidelines note that such factors should be examined in this manner for both primary and secondary outcomes, regardless of their explanatory power.

Leppink, O’Sullivan, and Winston [34] stress the importance of group-by-time interaction effects in studies with longitudinal outcomes for different groups, precisely the form of the present RCT data. Though not mandatory, several plausible hypotheses could account for such effects in these data. These include social network cross-pollination between intervention arms within close-knit communities, changes in macro-level socio-economic and political realities, and differences in (trial) educational adherence. Alosh et al. [2] likewise emphasize the crucial nature of subgroup analysis with RCT data without considering the longitudinal case.

To address the lack of standardized RCT sub-group analysis guidelines, Schandelmaier et al. [47] recently proposed an Instrument to assess the Credibility of Effect Modification Analyses (ICEMAN) for RCT data. By this instrument, the age-time-treatment interaction achieves high credibility, especially when considered with the aforementioned literature and FDA guidelines.

The generalizability of the Microclinic ("*Kanyakla*") Social Network Model for HIV/AIDS needs to be further examined. In particular, the issue of age and gender should be further examined in other settings since new infections disproportionately affect women and men at different ages [46]. While it has not been replicated in other locations for HIV/AIDS, the fact that clinical improvements have been induced in different study locations for other chronic conditions suggests there are generalizable social network health effects at play.

A further limitation of this study is the fact that it was implemented on a small Island community where people know each other. The Microclinic Social Network Model, by design, seeks to spread positive emotions, de-stigmatize, and improve behavior. A randomized trial to detect the effects of a social network-based educational program is bound to run into cross-pollination issues, whereby members of the same community, circle of friends, or family find themselves sharing advice, education, and support between intervention and controls. Importantly, we believe that such cross-pollination, while in some ways a limitation, actually fortifies our findings rather than diminishing them. This is based on the fact that it is more difficult, not less, to see an effect when there is cross-pollination. Despite these limitations, we believe our findings will be of interest to practitioners and policy-makers alike as they seek to integrate social network programming into precision HIV/AIDS prevention and monitoring strategies. Further efforts to study and personalize interventions to accommodate different ages, genders, socio-economic factors should be pursued.

Finally, the focus of the paper is on age. Effects by gender were not found to be significant. These two factors, and especially age, were chosen due to FDA guidelines suggesting that clinical trial analyses should perform effect modifications. While there may be other important factors, such as cultural beliefs, educational attainment, and general socio-economic status that might affect intervention outcomes, our study population was a homogenous island community and these variables were not readily available in the dataset utilized. Further qualitative and quantitative studies can explore possible connections between gender and treatment outcomes.

## Data Availability

The data that support the findings of this study are available here: https://datadryad.org/stash/dataset/doi:/10.7272/Q60V8B19.

## Abbreviations

HIV/AIDS: Human Immunodeficiency Virus/Aquired Immune Deficiency Syndrome
ART: Antiretroviral Therapy
